# Formation of Cell Membrane Component Domains in Artificial Lipid Bilayer

**DOI:** 10.1038/s41598-017-18242-9

**Published:** 2017-12-20

**Authors:** Ryugo Tero, Kohei Fukumoto, Toshinori Motegi, Miyu Yoshida, Michio Niwano, Ayumi Hirano-Iwata

**Affiliations:** 1 Department of Environmental and Life Sciences, Toyohashi University of Technology, Toyohashi, Aichi 441-8580 Japan; 2 Electronics-Inspired Interdisciplinary Research Institute, Toyohashi University of Technology, Toyohashi, Aichi 441-8580 Japan; 3 Laboratory for Nanoelectronics and Spintronics, Research Institute of Electrical Communication, Tohoku University, Sendai, Miyagi 980-8577 Japan; 4 Kansei Fukushi Research Institute, Tohoku Fukushi University, Sendai, Miyagi 989-3201 Japan; 5 Advanced Institute for Materials Research, Tohoku University, Sendai, Miyagi 980-8577 Japan; 6 Present Address: Division of Molecular Science, Faculty of Science and Technology, Gunma University, Kiryu, Gunma, 376-8515 Japan

## Abstract

The lipid bilayer environment around membrane proteins strongly affects their structure and functions. Here, we aimed to study the fusion of proteoliposomes (PLs) derived from cultured cells with an artificial lipid bilayer membrane and the distribution of the PL components after the fusion. PLs, which were extracted as a crude membrane fraction from Chinese hamster ovary (CHO) cells, formed isolated domains in a supported lipid bilayer (SLB), comprising phosphatidylcholine (PC), phosphatidylethanolamine (PE), and cholesterol (Chol), after the fusion. Observation with a fluorescence microscope and an atomic force microscope showed that the membrane fusion occurred selectively at microdomains in the PC + PE + Chol-SLB, and that almost all the components of the PL were retained in the domain. PLs derived from human embryonic kidney 293 (HEK) cells also formed isolated domains in the PC + PE + Chol-SLB, but their fusion kinetics was different from that of the CHO-PLs. We attempted to explain the mechanism of the PL-SLB fusion and the difference between CHO- and HEK-PLs, based on a kinetic model. The domains that contained the whole cell membrane components provided environments similar to that of natural cell membranes, and were thus effective for studying membrane proteins using artificial lipid bilayer membranes.

## Introduction

The lipid bilayer and membrane proteins in cell membranes constitute reaction fields for the transport of materials, signals, and energy in and out of cells through the membranes. The lipid bilayer is a structure of amphiphilic lipid molecules, which is self-assembled via hydrophilic and hydrophobic interactions; it provides an environment to membrane proteins, allowing them to retain their structure and functions. The movement of lipids and proteins is restricted in the direction perpendicular to the membrane; however, the molecules can diffuse laterally through lipid bilayers. This lateral diffusivity of the lipid bilayer is essential for the flexibility of lipid bilayer membranes (for example, membrane fusion and shape transformation) and for the formation of two-dimensional assemblies in lipid bilayer membranes, (for example, domains and clusters). The fusion of lipid bilayer membranes is a physiologically important process, especially for vesicular transportation^1^; it is also important in artificial lipid bilayer systems, such as free-standing bilayer lipid membranes (BLM), liposomes, and supported lipid bilayers (SLBs)^2^. For investigating membrane proteins using these artificial lipid bilayer systems, proteoliposomes (PLs) are fused with these artificial lipid bilayer membranes for reconstructing membrane proteins. It is necessary to understand the process of membrane fusion and the state of molecular distribution after the fusion to improve the efficiency of the reconstruction and to design and construct artificial lipid bilayer systems with natural lipids and proteins.

Membrane fusion is a phenomenon that has physical, chemical, and biological aspects^3–5^. The energy barrier for the fusion process is the contact between two bilayer membranes and the formation of an intermediate hemi-fusion state. Chanturiya *et al*. had reported that only 16% of vesicles in the vicinity of a BLM are in contact with the BLM, resulting in the exchange of lipid molecules^6^. Keidel *et al*. had estimated the energy barrier for the formation of the hemi-fusion state as ~10 k_B_ *T* ^7^. The addition of specific substances into the aqueous phases of the bilayers could induce the fusion of lipid bilayer membranes^8–11^. The energy barrier at the “contact” step originates from hydration repulsion. Electrostatic interactions mediated by multivalent cations or the perturbation of the hydration structure by the addition of hydrophilic polymers such as polyethylene glycol could contribute to overcoming the contact energy barrier and proceeding to membrane fusion. Physical pressures such as osmotic pressures and centrifugal force^12^ have been found to improve the efficiency of membrane fusion. At the hemi-fusion state, proximal leaflets of the bilayer membranes in contact form a negative curvature, whereas liposomes have a positive curvature. The addition of small organic molecule or the inclusion of cone-shaped lipids such as phosphatidylethanolamine and phosphatidylglycerol into the lipid bilayers could stimulate the formation of the hemi-fusion state by stabilizing the negative curvature^3,5,8^. During the fusion of synaptic vesicles, SNARE proteins are known to drive the contact and fusion of the lipid bilayer membranes^1,13,14^.

The distribution of membrane proteins and natural lipids from PLs after fusion with synthetic membranes is a key factor for the activity of membrane proteins. Domains and clusters of lipids and proteins play crucial roles in the reactions in lipid bilayers, as represented by the concept of rafts^15^. Membrane proteins retain their specific structure and functions due to the surrounding lipid molecules^16,17^. In order to ensure that the environment around membrane proteins is maintained similar to that of natural cell membranes, whole cell membrane fractions could be used for studying the membrane protein functions and protein-lipid interactions in artificial lipid bilayer systems^12,18,19^.

In a series of studies for recording channel currents, BLMs comprising phosphatidylcholine (PC), phosphatidylethanolamine (PE), and cholesterol (Chol) were used to retain the activity of ion channels such as Kv 11.1, Na_v_1.5, GABA_A_ receptors, and NADA receptors^12,19,20^. Channel current recording is considered a gold standard for studying the functions of ion channels at the molecular level; however it provides no information on the mechanism of PL fusion and the distribution of PL components. In this study, we investigated the mechanism of fusion of PLs derived from cultured cells and the distribution of the PL components, using SLBs.

## Results and Discussion

### Domains of cell membrane components in supported lipid bilayer

**Figure 1:**
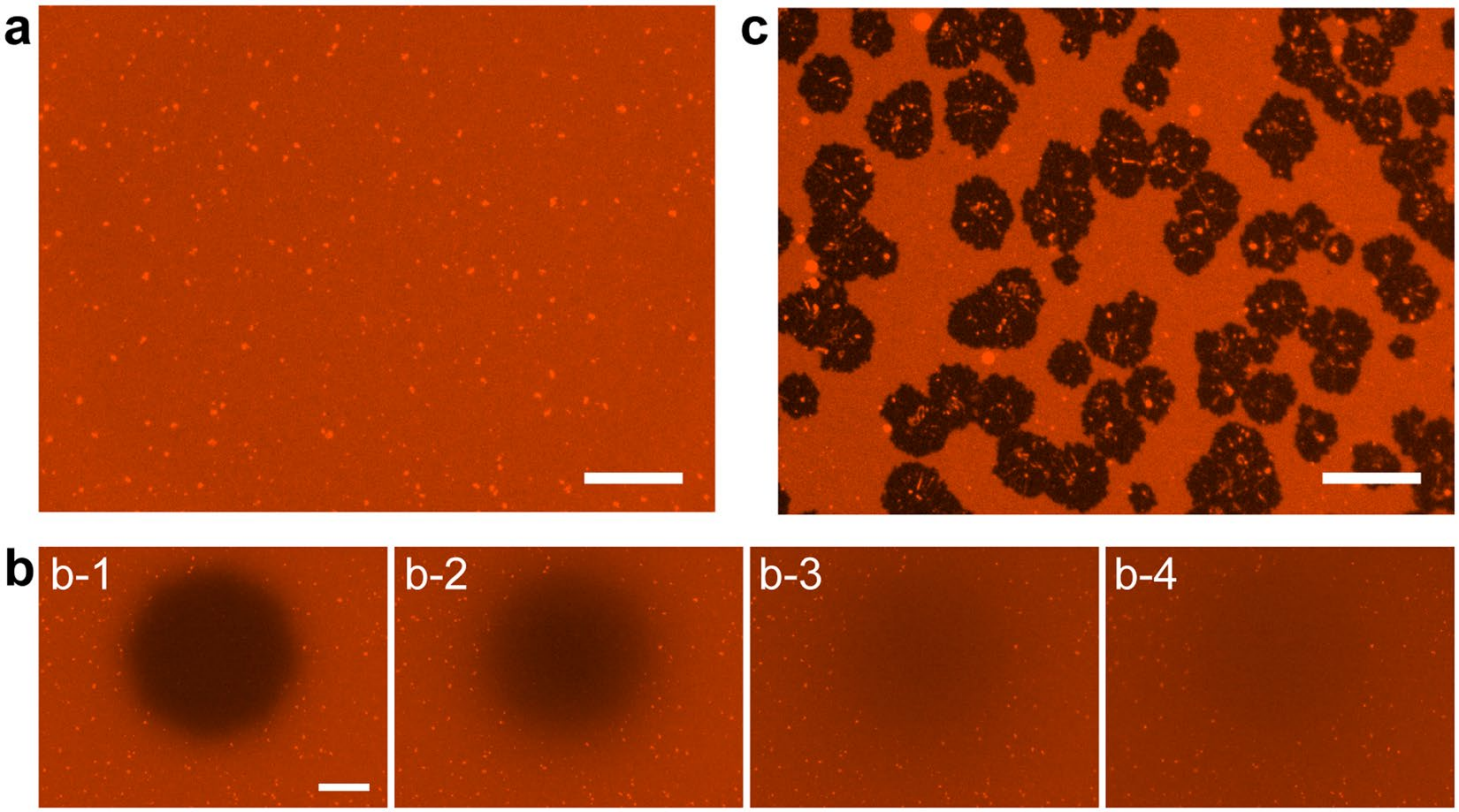
Fluorescence images of supported lipid bilayer (SLB) before and after fusion with proteoliposomes (PLs). (**a**) The SLB comprising phosphatidylcholine (PC), phosphatidylethanolamine (PE), and cholesterol (Chol) before the fusion of Chinese hamster ovary (CHO)-PL and photobleaching, and (**b**) its fluorescence recovery after photobleaching (FRAP) process: (b-1) 0 s, (b-2) 60 s, (b-3) 360 s, and (b-4) 600 s after the photobleaching. (**c**) PC + PE + Chol-SLB after fusion with CHO-PL. Scale bar = 20 µm.

**Figure 2:**
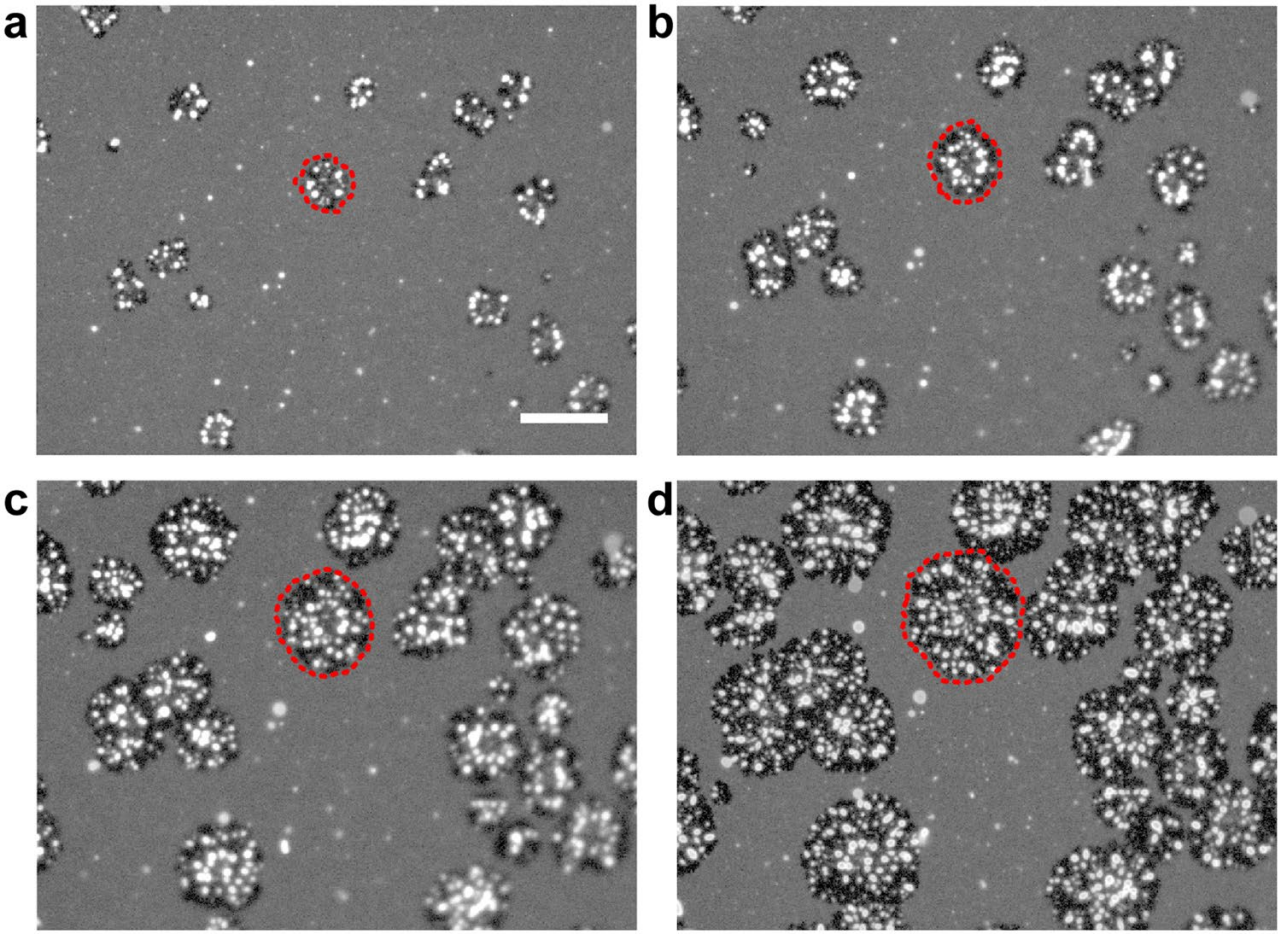
*In-situ* observation of the fusion of CHO-PL with the PC + PE + Chol-SLB at 37 °C. Snapshots were obtained from time-lapse fluorescence images at (**a**) 0 s, (**b**) 300 s, (**c**) 900 s, and (**d**) 2235 s after the sample temperature reached 37 °C. One of the growing PL-domains is marked with a red dotted circle. The images are shown in grayscale to facilitate visualization. Scale bar = 20 µm.

**Figure 3:**
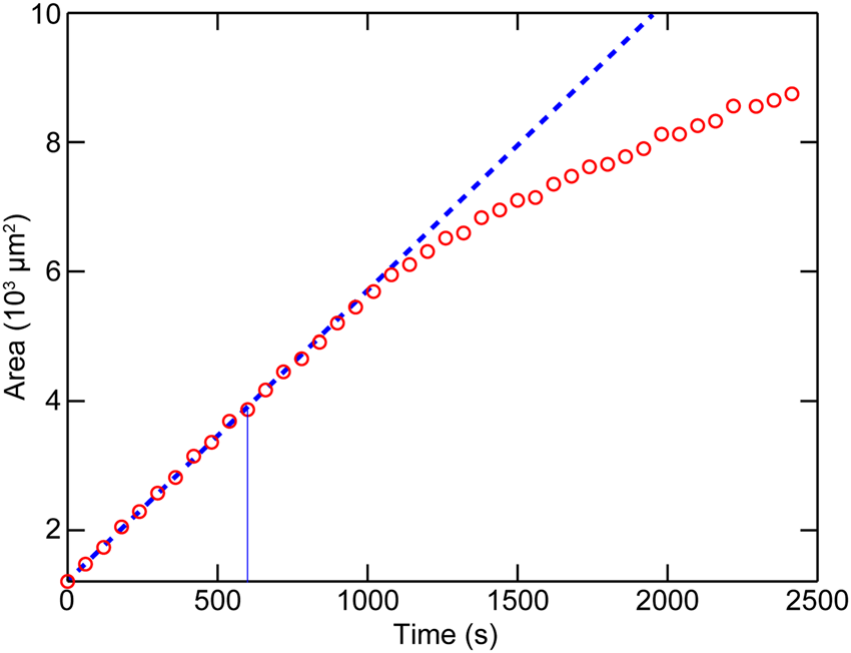
Time-dependence of the area of CHO-PL domains. Area of the CHO-PL (dark) domains in the view-field of Fig. 2 (140.8 × 105.6 µm^2^) was calculated from each time-lapse fluorescence image. Blue dotted line has a gradient of initial area increase rate 4.5 µm^2^/s, which was obtained from the linear fitting of the points at *t* = 0–600 s (indicated by blue line).

**Figure 4:**
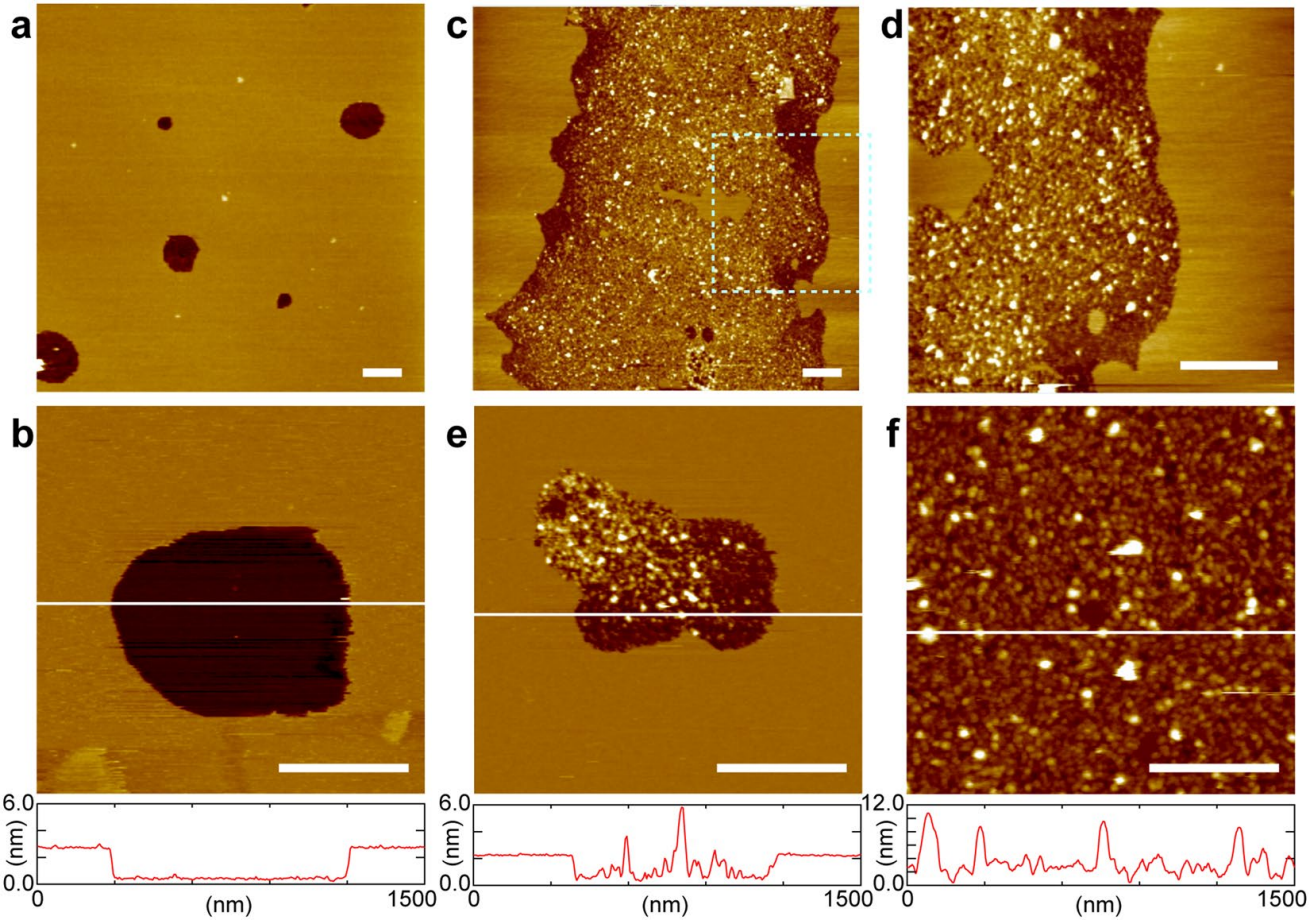
AFM topographies of PC + PE + Chol-SLB before and after fusion with CHO-PL, and CHO-PL-SLB. (**a**,**b**) Before the fusion of CHO-PL, (**a**) 5.0 × 5.0 µm^2^ and (**b**) 1.5 × 1.5 µm^2^, with the cross section profile at the white line. (**c**,**d**) After incubation with CHO-PL in the same conditions as Fig. 1b (40× diluted PL suspension, at 37 °C for 60 min), (**c**) 5.0 × 5.0 µm^2^, and (d) 2.0 × 2.0 µm^2^ (zoomed at the area of the dotted square in (**c**)). (**e**) After incubation with 100× diluted CHO-PL suspension at 37 °C for 30 min (1.5 × 1.5 µm^2^), with the cross section profile. (**f**) AFM topography (1.5 × 1.5 µm^2^) after the substrate without PC + PE + Chol-SLB was incubated in the CHO-PL suspension, with a cross section profile. Scale bar = 500 nm.

Figure 1 shows fluorescence images before and after the addition of Chinese hamster ovary (CHO)-PLs to PC + PE + Chol-SLB, which was prepared by the vesicle fusion method^21,22^ on a mica substrate. It showed uniform fluorescence intensity over almost the entire sample surface (Fig. 1a). Sparsely existing bright spots with the area fraction of ~1%, indicating unruptured vesicles on SLB^23^, were also observed. Figure 1b shows a series of fluorescence images during fluorescence recovery after photobleaching (FRAP)^24^. The fluorescence intensity of the bleached region recovered over time (Fig. 1b-1 to b-4) because of the lateral diffusion of lipid molecules. The results in Fig. 1a and b showed the formation of uniform, fluid, and continuous SLB. We added a suspension of CHO-PL to PC + PE + Chol-SLB, and incubated it at 37 °C for 60 min. The CHO-PL suspension was diluted to 40× , which contained 0.038 mg/mL proteins (See Materials and Methods for details). Figure 1c shows the PC + PE + Chol-SLB after incubation and washing. Dark domains were found in the PC + PE + Chol-SLB. We attributed these dark domains to components of the CHO-PLs that were fused with PC + PE + Chol-SLB, because the cell membrane of the cultured CHO cells was not stained with fluorescent dyes. The fluorescence intensity of the PC + PE + Chol-SLB around the PL-fused domain was almost the same as that before the fusion, indicating that the CHO-PL components were almost immiscible in the PC + PE + Chol-SLB. Domains containing the cell membrane components were formed in this SLB system.

We have described the fusion process of PL and the distribution of the PL-fused domains in the following sections. Figure 2 shows snapshots from the in-situ time-lapse observation of the fusion process of CHO-PL. The movie of the sequential images is shown as Supplementary Information (Movie S1). We added the CHO-PL suspension to PC + PE + Chol-SLB at the same concentration as that in Fig. 1b at 25 °C, and increased the temperature to 37 °C. The dark domains appeared (Fig. 2a) and expanded over time (Fig. 2b–d). Blebs of bright lipid bilayers then gradually appeared on the dark domains. Unstained CHO-PL fused with SLB, and squeezed out the PC + PE + Chol-bilayer containing dye-labelled lipids, which were observed as the bright blebs. The growth of the dark domains did not occur at 25 °C, and started around 35 °C when we increased the temperature from 25 °C to 37 °C. Majority of the unruptured vesicles on PC + PE + Chol-SLB were immobile at 37 °C and stayed out of the growth of the dark domains or bright blebs. In the absence of PL, PC + PE + Chol-SLB did not show any morphological change at 37 °C (Figure S1 in Supplementary Information). The blebs disappeared after the washing process to remove excess CHO-PL from the aqueous phase, but the dark domains remained as in Fig. 1b. We noted that the size of the PL-fused domains increased, but their number remained constant. This result indicated that specific sites for the membrane fusion existed in the SLB.

Time dependence of the area of the dark PL-fused domains in the view field in Fig. 2 (140.8 × 105.6 µm^2^) is shown in Fig. 3. The areas were obtained from the time-lapse fluorescence images, and plotted against time (*t*). Note that the *t* = 0 s corresponds to the time sample temperature reached to 37 °C. Figure 3 shows that the area increases linearly until *t* ~ 1000 s. The increase rate obtained from the linear fitting at t = 0–600 s was 4.5 µm^2^/s (blue dotted line in Fig. 3). If we approximate the PL diameter (*d*) as ~400 nm because the diameter of CHO-PL distributed around 200–600 nm, the rate of the PL fusion per unit area (µm^2^) is estimated to be 6 × 10^−4^ PLs s^−1^ µm^−2^. The initial fusion rate until *t* ~ 1000 s was constant, thus independent of the area of the PL-fused domain. It means that the PL components fused to SLB did not work as the site for the membrane fusion.

### Fusion site in PC + PE + Chol-SLB

We observed microscopic structures of PC + PE + Chol-SLB before and after fusion with CHO-PL, using AFM. Figure 4a and b show the AFM topography of PC + PE + Chol-SLB obtained with a cantilever, at a spring constant of 0.1 N/m. Depressed regions were observed with average diameter and area fraction of 375 nm and 4.9%, respectively. The depth of the depressed region was ~1.9 nm. The depressed regions were not holes in the SLB, but domains of a thinner lipid bilayer than the surrounding region: the thickness of PC + PE + Chol-SLB was 5.4 nm (Figure S2, Supplementary Information), which is consistent with that of a single lipid bilayer (around ~5 nm), according to previous AFM studies^23,25^. The thickness of the bilayer at the depression domain, 3.5 nm, seems small as the thickness of a lipid bilayer, but because of the compression of lipid bilayers by the cantilever tip, the height difference between two lipid domains with different elasticity (e.g. liquid crystalline [L_α_] and gel phases, and liquid ordered [L_o_] and liquid disordered [L_d_] phases) may be enhanced in AFM topographies obtained in the conventional tapping mode^23,25,26^. Previous AFM force spectroscopy studies indicate that even an applied force of ~0.1 nN compresses a lipid bilayer in L_α_ phase ~1 nm^27,28^. PC + PE + Chol-SLB showed uniform fluorescence intensity in the optical microscope (Fig. 1a and b), but contained submicron domains (Fig. 4a and b) which are not resolved with a conventional optical microscopy.

Figure 4c and d show the AFM topographies of PC + PE + Chol-SLB after incubation in the same conditions as Fig. 1c (with 40× diluted CHO-PL suspension [0.038  mg/mL protein] at 37 °C for 60  min). Many protrusions were found in the flat SLB. The regions with the protrusions were separated from the flat regions. The height of the protrusions varied from ~4  nm to 25  nm. The CHO-PL used in the present study was a crude membrane fraction without purification; therefore, it contained all membrane proteins and glycolipids that were observed as protrusions of various sizes. We had attributed the region with these protrusions to the dark region in the fluorescence images in Figs 1c and 2. Careful observation of the distribution of the protrusions revealed a low density of the protrusions near the boundary (Fig. 4d). Additionally, the depression domains (Fig. 4a) disappeared in the flat region after fusion with CHO-PL. Figure 4e shows the AFM topography of PC + PE + Chol-SLB incubated in CHO-PL suspension of a lower concentration (0.012  mg/mL of protein) for a shorter time (30  min) at 37 °C, than Fig. 4c and d. These images revealed that the fusion of CHO-PL occurred preferentially at the depression domains.

We also show the AFM topography of CHO-PL-SLB in Fig. 4f: the mica substrate without PC + PE + Chol-SLB was incubated in the same condition as Fig. 4c and d. Protrusions with a similar range of size to Fig. 4d and e were observed. This image proves that the protrusions in Fig. 4c–e were components in CHO-cell membranes. The morphology of the SLB out of the PL-fused domain remained flat in the AFM topographies after the incubation with CHO-PL (Fig. 4c–e), as with the fluorescence images in Figs 1c and 2. We estimated the approaching frequency of PL to SLB to be ~80 PLs μm^−2^ per 60 min from the diffusion constant of PL (*d* ~ 400 nm) in a bulk aqueous solution at 37 °C which was 1.6 µm^2^ s^−1^ based on the Stokes-Einstein equation (details are in Supplementary Information). We did not find trace of disruption in the SLB region out of the PL-fused domains in the AFM topographies (Fig. 4) or in the fluorescence images (Figs 1 and 2), although PLs approached to SLB 80 times per μm^2^ during the incubation with CHO-PL for 60 min. Fusion of PL to PC + PE + Chol-SLB selectively occurred at the depression domain.

### Fusion of HEK 293 PLs

**Figure 5:**
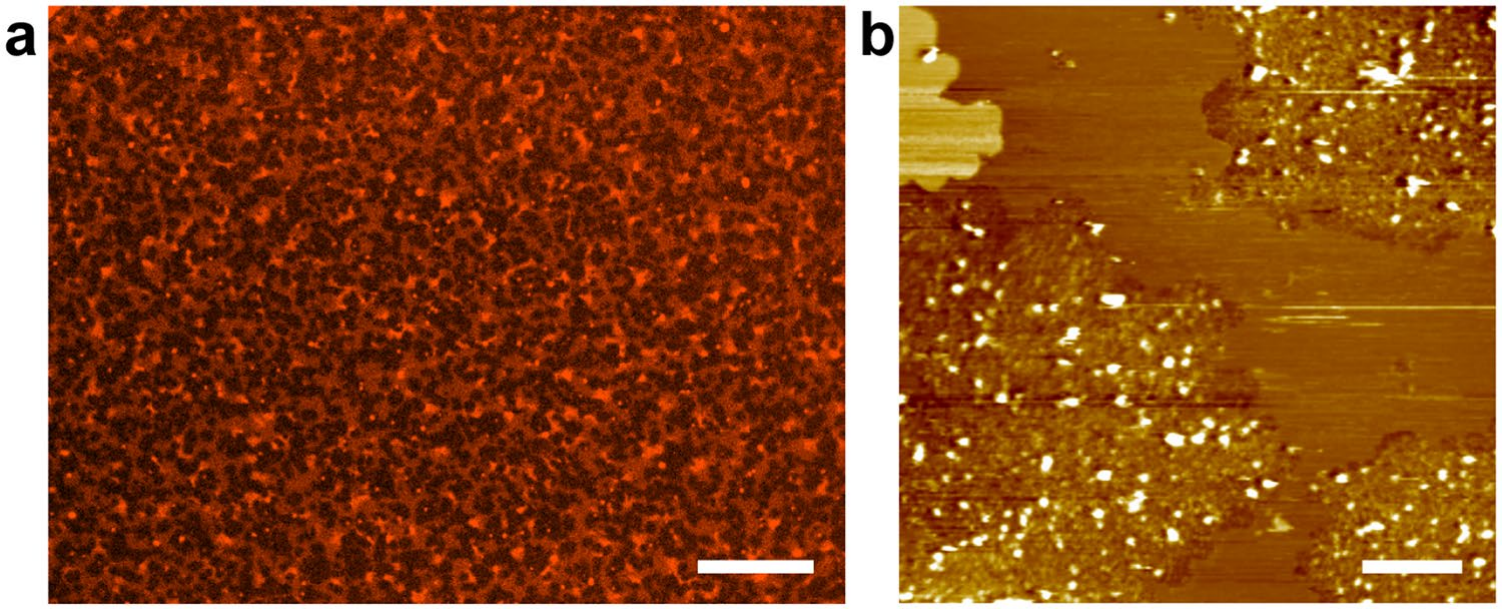
PC + PE + Chol-SLB after the fusion of HEK-PL. PC + PE + Chol-SLB was incubated with HEK-PL at similar condition as shown in Fig. 1b. (**a**) Fluorescence image. Scale bar = 20 µm. (**b**) AFM topography (5.0 × 5.0 µm^2^). Scale bar = 500 nm.

The mechanism of the fusion of PLs was highly dependent on the cell species from which it was extracted. Figure 5 shows a fluorescence image and AFM topography of PC + PE + Chol-SLB after incubation with PLs extracted from HEK293 cells (HEK-PL). The concentration of PLs and the incubation conditions were similar to those shown in Fig. 1b, with 40× diluted PL suspension (0.034 mg/mL protein) at 37 °C for 60 min. The components of HEK-PLs were isolated from PC + PE + Chol-SLB and observed as dark domains in the fluorescence image (Fig. 5a), similar to those of CHO-PLs (Fig. 1). However, the size and distribution of the PL-fused dark domains were different for the two PLs. A large number of small dark domains were found to be distributed over the SLB. AFM topography (Fig. 5b) also revealed domains with protrusions after fusion with HEK-PLs.

### Mechanism of PL fusion

The results of our experiments showed that the domains of CHO and HEK cell membrane components were formed in the SLB after fusion with the PLs. The mechanism and kinetics of the PL fusion with SLB was strongly associated with the microdomains in the SLB and the species of cells from which the PLs were derived. The key points of the fusion event, as indicated by the experimental results, are summarised below:

- Lipid molecules laterally diffused through the PC + PE + Chol-SLB.
- The fusion of PLs occurred at specific sites (depression microdomains) in the SLB.
- The depression microdomains disappeared after the fusion of PL with the PC + PE + Chol-SLB region (outside the PL-fused domains). In the PL-fused domains, the density of the protrusions was lower at the peripheral regions than the central region.
- The rate of PL fusion was constant and independent of the area of the PL-fused domains.
- The size and density of the PL-fused domains depended on the species of cells from which the PLs were extracted.

**Figure 6:**
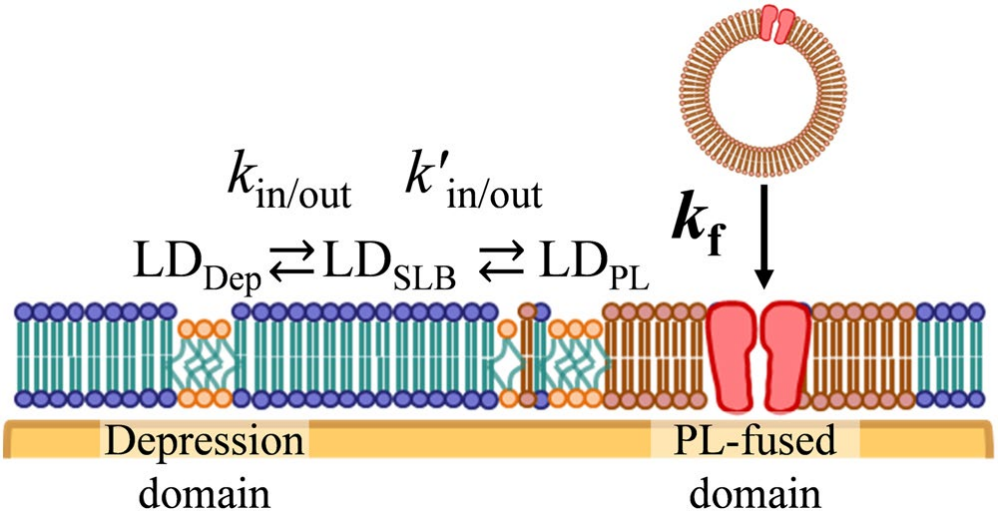
Kinetic model of PL fusion with PC + PE + Chol-SLB. LD indicates lipid components in the depression domains, and subscripts Dep, PL, and SLB represent LDs in the corresponding depression domains, PL-fused domains, and the SLB regions outside these domains, respectively. *k*_f_ is the kinetic constant of the PL fusion (Eq. (1)), and *k*_in/out_ and *k*′_in/out_ are the kinetic constants of diffusion of LD into/out of the depression domains and PL-fused domains in SLB, respectively.

We proposed a mechanism for membrane fusion to SLB based on these experimental results (Fig. 6). The lipid components of the depression microdomains (LD) in PC + PE + Chol-SLB have higher affinity to the PLs. It is to be noted that the domains and the outer regions of SLB were in equilibrium because of the lateral diffusion of lipids (Fig. 1b), although the domains were immobile and constant in an SLB^22^. The PLs fused to the microdomains and their components were miscible, while the PL-fused domains were immiscible, in the surrounding PC + PE + Chol-SLB. After the introduction of the PL-fused domains into the SLB, the lipids in the other depression microdomains were absorbed into the PL-fused domain through diffusion in the PC + PE + Chol-SLB. The regions with absorbed lipids existed at the periphery of the PL-fused domains until the fusion of another PL, as the majority of membrane proteins do not diffuse into the PL-fused domain, as observed in the AFM topographies (Fig. 4). The disappearance of the depression microdomains proceeded competitively to the PL fusion. When PL fusion proceeds at a higher pace, more PL domains appear before the microdomains are absorbed to other PL domains; similar results were obtained for HEK-PLs as well (Fig. 5).

Kinetics of the fusion and diffusion in SLB are described below:

1 $$P{L}_{aq}+L{D}_{Dep}\mathop{\longrightarrow }\limits^{{k}_{f}}P{L}_{SLB}$$

2 $$L{D}_{Dep}\begin{array}{c}\mathop{\longrightarrow }\limits^{{k}_{out}}\\ \mathop{\longleftarrow }\limits_{{k}_{in}}\end{array}L{D}_{SLB}\begin{array}{c}\mathop{\longrightarrow }\limits^{k{\text{'}}_{in}}\\ \mathop{\longleftarrow }\limits_{k{\text{'}}_{out}}\end{array}L{D}_{PL}.$$

*PL*_aq_ and *PL*_SLB_ represent PLs in the aqueous phase before the fusion and PLs in the PL-fused domain of SLB, respectively. LD represents the lipid components that form the depression domains, and *LD*_Dep_, *LD*_PL_, and *LD*_SLB_ represent those in the depression domains, PL-fused domains, and the SLB regions outside these domains, respectively. The PL fusion occurs irreversibly, with the rate constant *k*_f_. The LDs are in equilibrium between the depression domains and the surrounding SLB at equilibrium constant *K* = *k*_out_/*k*_in_ before the PL fusion. After the PL fusion, the LD components are exchanged between the depression domains and the PL-fused domains through the surrounding SLB region following the concentration gradient, until equilibrium is attained. Therefore, equation (2) may be simplified as:

3 $$L{D}_{Dep}\begin{array}{c}\mathop{\longrightarrow }\limits^{{k}_{1}}\\ \mathop{\longleftarrow }\limits_{{k}_{2}}\end{array}L{D}_{PL}.$$

### Kinetic simulation of domain size and density

We calculated the size and density of domains using these kinetic modes. In each step of the calculation, the fusion of PL to the domains and the diffusion of LDs between the domains proceeded as follows. The fusion of PL occurs at the domains with a probability per unit area of *P*_f_ = *k*_f_ *ρ C*, where *ρ* is the density of PL in the suspension and *C* is the concentration ratio of LDs in the domain. The total amount of LD is constant and so, the fusion probability in the total area is also constant, as shown in Fig. 3. When PL fusion occurs, the area of the domain increases by the area of a PL (*A*_PL_), and *C* is decreased. After the detection of PL fusion, the LDs move between the PL-free domains and the PL-fused domains, depending on the concentration gradient (Eq. (3)).

**Figure 7:**
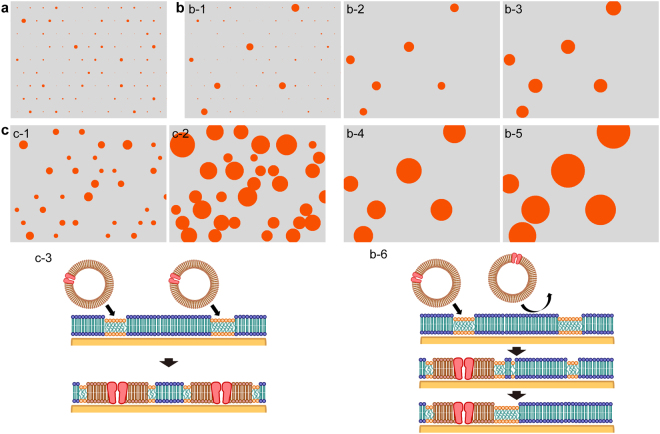
Growth of PL domains depending on *k*_f_. (**a**) The initial state. (**b**) Time course of the kinetic simulation with *k*_f_ = 0.0002 after (b-1) 50 steps, (b-2) 100 steps, (b-3) 300 steps, (b-4) 900 steps and (b-5) 1800 steps, and (b-6) a schematic of the fusion of PL and the disappearance of a depression domain. (**c**) Time course of the kinetic simulation with *k*_f_ = 0.02 after (c-1) 3 steps and (c-2) 20 steps, and (c-3) a schematic of the PL fusion with depression domains.

At the initial state, domains with random sizes settled as shown in Fig. 7a. We set the area fraction of the domains to 4%, average domain area to 10 px^2^ and *A*_PL_ to 50 px^2^: the ratio between the area of the depression domains (Fig. 4a) with average diameter of 375 nm and that of PL with diameter of 400 nm is around 1:5. After the PLs fused with some of the domains, the area of these domains increased by the areas of fused PL and by the inflow of the LD components (Fig. 7b-1). The domains that did not fuse with PLs gradually shrunk and disappeared because of the efflux of LD components (Fig. 7b-2). The flow of LD components attained equilibrium, but the existing domains kept growing because of the PLs fused to the LD components in them (Fig. 7b-3 to b-5). The result of this simulation replicated the fusion of CHO-PLs shown in Figs 2 and 3 (Fig. 7b-6). The movie of the sequential images is shown as Supplementary Movie S2. Higher values of *k*_f_ indicate that PL fusion occurred at more domains before the LD components effused completely (Fig. 7c, and Supplementary Movie S3). The most dominant physical parameter in this process is the rate of fusion of PL to the microdomains.

The microdomains in which PL fusion occurred were formed in the PC + PE + Chol-SLB containing 33 mol% of cholesterol. The PC and PE agents used in this study were extracted from chicken egg. The major acyl chains in these agents are oleoyl and palmitoyl groups, but various acyl groups with different lengths and degrees of unsaturation are included^29,30^. Generally, in a mixture of PCs with at least one unsaturated acyl chain, phase separation (to L_o_ and L_d_ phases) occurs in a PC with saturated acyl chains and cholesterol^31,32^. The former phase is thicker due to the higher packing of acyl chains induced by cholesterol than the latter. The latter mainly consists of lipids with unsaturated acyl chains and it therefore more fluid than the former. Previous studies have shown that cholesterol has a lower affinity to unsaturated acyl chains than to saturated ones^31–33^, and that it has lower affinity and miscibility to phosphatidylethanolamine than to phosphatidylcholine^33–35^. Generally in chicken eggs, PE tends to have more unsaturated acyl chains than PC^29,30^. Hence, it is reasonable to surmise that the depression domains are rich in PE and poor in cholesterol compared to the surrounding regions. It is known that PE contributes to the fusion of lipid bilayer membranes by providing a negative curvature at the hemi-fusion state^3,5,8^. Fluid domains rich in PE and poor in cholesterol might thus facilitate fusion.

Lipid molecules serve as a matrix, allowing membrane proteins to retain their structure and functions^16,17^. In artificially prepared PC + PE + Chol-SLB, the membrane proteins and lipids are stored in PL-fused domains, and therefore, they are maintained in an environment similar to that in living cells. Fusion events in artificial lipid bilayer systems occurs only if the two lipid bilayer membranes are in the fluid phase; thus, the components in the two membranes intermix after the fusion^6,13,36,37^. Further studies are needed to understand the reason for the immiscibility of PL components in SLB, even though PC, PE and Chol are the major components of CHO and HEK 293 cell membranes. The isolated PL-fused domains in this study would be a useful platform for future studies, as they contain the components of the whole cell membrane.

### Summary and Conclusions

PLs derived from CHO and HEK 293 cells formed isolated domains in an artificial PC + PE + Chol-SLB. In situ fluorescence microscope observation showed that the PL domains formed in the PC + PE + Chol-SLB, squeezing out the excess SLB. In the AFM topographies, the components of PLs were observed as isolated domains with protrusions; however, such protrusions were not found in the regions of PC + PE + Chol-SLB. The heterogeneous conditions in the PC + PE + Chol-SLB, the microdomains, and their surrounding regions play different roles in the fusion and isolation of PLs in SLB. The microdomains had an affinity to PL and served as a selective fusion site, while the surrounding region was immiscible to PL, resulting in the PL components forming isolated domains. The CHO-PL and HEK-PL showed differences in the size and distribution of domains after the fusion, although both formed similar isolated domains in the SLB. The kinetic model and the simulation revealed that the difference was mainly because of the initial rate of the PL fusion to microdomains, assuming that the components of the microdomains laterally diffused through the SLB. The isolated PL-fused domains maintains a lipid membrane environment similar to that in natural cell membranes, and is therefore valuable for the studies using artificial lipid bilayers.

## Materials and Methods

### Materials

L-α-phosphatidylcholine (PC) (chicken egg) and L-α-phosphatidylethanolamine (PE) (chicken egg) were purchased from Avanti Polar Lipids (Alabaster, USA) and a dye-labelled lipid β-BODIPY® 530/550 C_5_-HPC (BODIPY-PC) was purchased from Invitrogen (currently Thermo Fisher Scientific, Eugene, USA). They were used without further purification. Cholesterol (Chol) and the chemicals for a buffer solution (120 mM KCl and 10 mM HEPES/KOH [pH 7.2]) were purchased from Wako Pure Chemicals Industries (Osaka, Japan). Chol was recrystallized thrice from methanol.

### Preparation of lipid vesicles and SLB

The stock solutions of the lipids in organic solvents were mixed at a ratio of PC:PE:Chol:BODIPY-PC = 7:1:2:0.05 (w/w) (molar ratio of 0.58:0.09:0.33:0.0035) in a glass vial. The solution was dried under a stream of nitrogen and stored in vacuum for >6 h. The lipid film was then agitated in the buffer solution. A suspension of unilamellar vesicles was prepared by subjecting the solution to five freeze-thaw cycles, extrusion through a 100-nm polycarbonate filter, and sonication. SLB was formed on a piece of freshly cleaved Muscovite mica after incubation in the vesicle suspension at 37 °C for 1 h. The excess vesicles were removed after the incubation by exchanging the vesicle suspension with the buffer solution ten times.

### Extraction of plasma membrane fraction

The CHO cell line and the HEK 293 cells were obtained from the Channelopathy Foundation (Basel, Switzerland) and Anaxon AG (Berne, Switzerland), respectively. The CHO cell line was maintained in a HAM/F-12 medium supplemented with 10% foetal bovine serum (FBS), 1% penicillin/streptomycin and 100 μg/ml hygromycin B (GIBCO, Thermo Fisher Scientific). HEK293 cells were maintained in a DMEM/GlutaMAX medium supplemented with 10% FBS and 1% penicillin/streptomycin under an antibiotic pressure of 100 μg/ml geneticin (GIBCO). PLs were obtained from these cell lines as membrane fractions, using procedures described in previous reports^12,19^. Briefly, the cells were rinsed with HBSS (GIBCO) and then scraped off into 200 mM NaCl, 33 mM NaF, 10 mM EDTA, 50 mM HEPES (pH 7.4 with NaOH), and protease inhibitors (100 μM phenylmethylsulfonyl fluoride, 1 μg/ml pepstatin A, and 1 μg/ml leupeptin). The cells were homogenized and spun at 1,500 × *g* for 10 min. The membrane fractions were pelleted from the low-speed supernatants by centrifugation at 157,000 × *g* for 1 h and resuspended in 120 mM KCl solution containing 10 mM HEPES (pH 7.2 with KOH). The concentration of the PL suspension was evaluated with the amount of proteins in the PLs, which was 1.2–1.5 mg/mL for both CHO- and HEK-PL suspensions. The size of CHO-PL distributed around 200–600 nm in diameter^12^. The PL suspension was diluted 40–100 times when it was added to SLB.

### Observation of SLB

The SLB samples were observed with an AFM (PicoPlus 5500, Keysight Technologies, Inc., Santa Rosa, USA, formerly Molecular Imaging, Corp.) and an epi-fluorescence microscope (BX51WI, Olympus, Tokyo, Japan) in the buffer solution at 25 °C unless otherwise noted. AFM topographies were obtained in the acoustic AC mode (tapping mode) using a Si_3_N_4_ cantilever with a spring constant of 0.1 N/m (BL-AC40TS, Olympus). We obtained AFM topographies at least five different positions of a sample to calculate average area fractions of domains and to confirm the generality of the observed phenomena. Fluorescence images were obtained using a high-pressure mercury lump, a mirror unit (U-MWIG3, with a long-pass emission filter >575 nm) and neutral density (ND) filters and recorded with a CCD camera (DS-Qi1Mc, Nikon, Tokyo, Japan). Pixel size was 0.22 µm/px with a 60× water-immersion lens (LUMPlan FL 60×, NA = 1.00), and the spatial resolution at the wavelength of 580 nm was approximately 350 nm based on the Rayleigh criterion. Area fractions of the domains in the fluorescence images and AFM topographies were obtained with Image J (NIH, USA, https://imagej.nih.gov/ij/) and SPIP (Image Metrology A/S, Hørsholm, Denmark), respectively.

### Kinetic simulation

At the initial state, 108 domains with random area values with normal distribution were settled in a field of 600 px × 450 px. The average size of the domains used in this study was 10 px^2^ corresponding to the area fraction of 4%. The area of the domains were varied through the following steps. (1) Fusion of PL was detected for each pixel of each domain with a probability of *P*_f_. (2) After the detection for all the domains, the area of each domain was increased by the area of PL (*A*_PL_ = 50 px^2^) by the frequency of fusion in (1). (3) The component of the domains (LD) was increased in every domain to which PL fused at least once, in proportion to the relative equilibrium constant *K*, the density of PL in the domain and the domain perimeter. *K* was an arbitrary parameter and set to 0.01. (4) The same amount as the sum of LD in (3) was removed from the domains to which PL had never fused, in proportion to their area. The domain was discarded if its area became less than 1 px^2^. The steps (1–4) were repeated, and the domains were redrawn with the new area after each cycle. Calculation and visualization were performed with Igor Pro software (Wavemetrics, Portland, USA).

## Electronic supplementary material

Supplementary Information

Movie S1

Movie S2

Movie S3

## References

[1] Rothman, J. E. The Principle of Membrane Fusion in the Cell (Nobel Lecture). *Angew. Chemie - Int. Ed*. **53**, 12676–12694 (2014). 10.1002/anie.201402380 25087728

[2] Hirano-Iwata A, Niwano M, Sugawara M (2008). The design of molecular sensing interfaces with lipid-bilayer assemblies. TrAC Trends Anal. Chem..

[3] Chernomordik LV, Kozlov MM (2008). Mechanics of membrane fusion. Nat. Struct. Mol. Biol..

[4] Sakuma Y, Imai M, Stano P, Mavelli F (2015). From Vesicles to Protocells: The Roles of Amphiphilic Molecules. Life.

[5] Yamazaki M, Tamba Y (2005). The Single GUV Method for Probing Biomembrane Structure and Function. e-J. Surf. Sci. Nanotechnol..

[6] Chanturiya A, Chernomordik LV, Zimmerberg J (1997). Flickering fusion pores comparable with initial exocytotic pores occur in protein-free phospholipid bilayers. Proc. Natl. Acad. Sci..

[7] Keidel A, Bartsch TF, Florin E-L (2016). Direct observation of intermediate states in model membrane fusion. Sci. Rep..

[8] Mondal Roy S, Sarkar M (2011). Membrane Fusion Induced by Small Molecules and Ions. J. Lipids.

[9] Tanaka T, Yamazaki M (2004). Membrane Fusion of Giant Unilamellar Vesicles of Neutral Phospholipid Membranes Induced by La^3+^. Langmuir.

[10] Lentz BR, Lee J (1999). Poly(ethylene glycol) (PEG)-mediated fusion between pure lipid bilayers: a mechanism in common with viral fusion and secretory vesicle release? (Review). Mol. Membr. Biol..

[11] Proux-Delrouyre V, Laval J-M, Bourdillon C (2001). Formation of Streptavidin-Supported Lipid Bilayers on Porous Anodic Alumina: Electrochemical Monitoring of Triggered Vesicle Fusion. J. Am. Chem. Soc..

[12] Hirano-Iwata A (2016). Reconstitution of Human Ion Channels into Solvent-free Lipid Bilayers Enhanced by Centrifugal Forces. Biophys. J..

[13] Fix M (2004). Imaging single membrane fusion events mediated by SNARE proteins. Proc. Natl. Acad. Sci..

[14] Liu T, Tucker WC, Bhalla A, Chapman ER, Weisshaar JC (2005). SNARE-Driven, 25-Millisecond Vesicle Fusion In Vitro. Biophys. J..

[15] Lingwood D, Simons K (2010). Lipid Rafts As a Membrane-Organizing Principle. Science.

[16] Lee AG (2004). How lipids affect the activities of integral membrane proteins. Biochim. Biophys. Acta.

[17] Jensen MØ, Mouritsen OG (2004). Lipids do influence protein function-the hydrophobic matching hypothesis revisited. Biochim. Biophys. Acta.

[18] Hsia C-Y, Chen L, Singh RR, DeLisa MP, Daniel S (2016). A Molecularly Complete Planar Bacterial Outer Membrane Platform. Sci. Rep..

[19] Oshima A (2013). Reconstitution of human ether-a-go-go-related gene channels in microfabricated silicon chips. Anal. Chem..

[20] Hirano A, Sugawara M, Umezawa Y, Uchino S, Nakajima-Iijima S (2000). Evaluation of agonist selectivity for the NMDA receptor ion channel in bilayer lipid membranes based on integrated single-channel currents. Biosens. Bioelectron..

[21] Mingeot-Leclercq M-P, Deleu M, Brasseur R, Dufrêne YF (2008). Atomic force microscopy of supported lipid bilayers. Nat. Protoc..

[22] Tero R (2012). Substrate Effects on the Formation Process, Structure and Physicochemical Properties of Supported Lipid Bilayers. Materials.

[23] Tero R, Ujihara T, Urisu T (2008). Lipid bilayer membrane with atomic step structure: supported bilayer on a step-and-terrace TiO_2_(100) surface. Langmuir.

[24] Axelrod D, Koppel DE, Schlessinger J, Elson E, Webb WW (1976). Mobility measurement by analysis of fluorescence photobleaching recovery kinetics. Biophys. J..

[25] Goksu EI, Vanegas JM, Blanchette CD, Lin W-C, Longo ML (2009). AFM for structure and dynamics of biomembranes. Biochim. Biophys. Acta.

[26] Yokota K, Toyoki A, Yamazaki K, Ogino T (2014). Behavior of raft-like domain in stacked structures of ternary lipid bilayers prepared by self-spreading method. Jpn. J. Appl. Phys..

[27] Connell SD, Smith DA (2006). The atomic force microscope as a tool for studying phase separation in lipid membranes (Review). Mol. Membr. Biol..

[28] Gumí-Audenis, B. *et al*. Structure and nanomechanics of model membranes by atomic force microscopy and spectroscopy: Insights into the role of cholesterol and sphingolipids. *Membranes (Basel)*. **6** (2016). 10.3390/membranes6040058 PMC5192414 27999368

[29] Pacetti D, Boselli E, Hulan HW, Frega NG (2005). High performance liquid chromatography-tandem mass spectrometry of phospholipid molecular species in eggs from hens fed diets enriched in seal blubber oil. J. Chromatogr. A.

[30] Shinn S, Liyanage R, Lay J, Proctor A (2014). Improved fatty acid analysis of conjugated linoleic acid rich egg yolk triacylglycerols and phospholipid species. J. Agric. Food Chem..

[31] Almeida PFF (2009). Thermodynamics of lipid interactions in complex bilayers. Biochim. Biophys. Acta.

[32] Quinn PJ, Wolf C (2009). The liquid-ordered phase in membranes. Biochim. Biophys. Acta.

[33] Niu S-L, Litman BJ (2002). Determination of Membrane Cholesterol Partition Coefficient Using a Lipid Vesicle–Cyclodextrin Binary System: Effect of Phospholipid Acyl Chain Unsaturation and Headgroup Composition. Biophys. J..

[34] Huang J, Buboltz JT, Feigenson GW (1999). Maximum solubility of cholesterol in phosphatidylcholine and phosphatidylethanolamine bilayers. Biochim. Biophys. Acta.

[35] Van Dijck PWM (1979). Negatively charged phospholipids and their position in the cholesterol affinity sequence. Biochim. Biophys. Acta.

[36] Wessels L, Elting MW, Scimeca D, Weninger K (2007). Rapid membrane fusion of individual virus particles with supported lipid bilayers. Biophys. J..

[37] Dewa T (2006). Lateral organization of a membrane protein in a supported binary lipid domain: direct observation of the organization of bacterial light-harvesting complex 2 by total internal reflection fluorescence microscopy. Langmuir.

