# Supplementary material for: Formation of Cell Membrane Component Domains in Artificial Lipid Bilayer
Source: Sci Rep. 2017 Dec 20;7:17905. doi: 10.1038/s41598-017-18242-9 (PMC5738377; doi:10.1038/s41598-017-18242-9)
Supplement: Supplementary file 1 — Supplementary Information [file 41598_2017_18242_MOESM1_ESM.pdf]

Supplementary Information

Formation of Cell Membrane Component Domains in Artificial Lipid Bilayer

Ryugo Tero[1,2,*], Kohei Fukumoto[1], Toshinori Motegi[2,†], Miyu Yoshida[3], Michio Niwano[3,4], and Ayumi Hirano-Iwata[3,5].

[1] Department of Environmental and Life Sciences, Toyohashi University of Technology, Toyohashi, Aichi 441-8580, Japan.

[2] Electronics-Inspired Interdisciplinary Research Institute, Toyohashi University of Technology, Toyohashi, Aichi 441-8580, Japan.

[3] Laboratory for Nanoelectronics and Spintronics, Research Institute of Electrical Communication, Tohoku University, Sendai, Miyagi, 980-8577, Japan.

[4] Kansei Fukushi Research Institute, Tohoku Fukushi University, Sendai, Miyagi, 989-3201, Japan.

[5] Advanced Institute for Materials Research, Tohoku University, Sendai, Miyagi 980-8577, Japan.

[†] Present Address: Division of Molecular Science, Faculty of Science and Technology, Gunma University, Kiryu, Gunma 376-8515, Japan

[*] Corresponding author: tero@tut.jp (R.T.)

Abbreviations

CHO: Chinese hamster ovary

PL: proteoliposome

PC: phosphatidylcholine (from chicken egg)

PE: phosphatidylethanolamine (from chicken egg)

Chol: cholesterol

SLB: supported lipid bilayer

Additional Data

In-situ observation of the fusion of CHO-PL with the PC+PE+Chol-SLB at 37 ºC.

Movie S1. The movie of the sequential time-lapse images, from which snapshots in Fig. 2 were obtained. The time after the sample temperature reached to 37 ºC is inserted.

PC+PE+Chol-SLB at 37 ºC without CHO-PL.

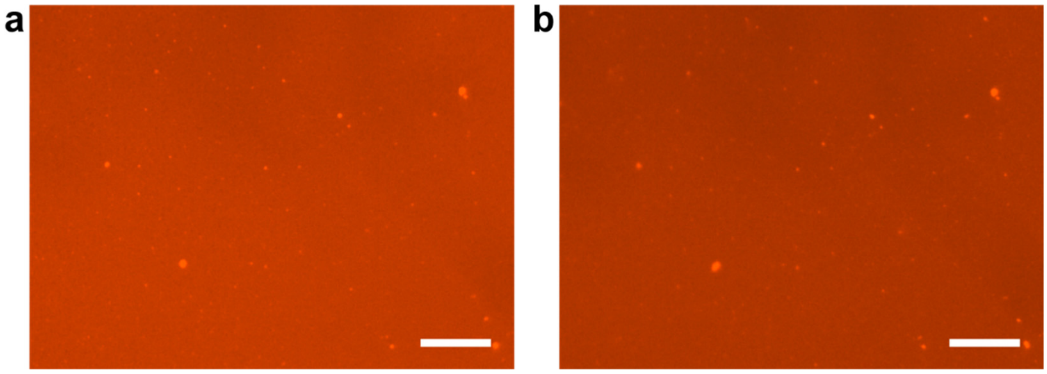

Figure S1. Temperature independence of PC+PE+Chol-SLB. Fluorescence images of the same position of a PC+PE+Chol-SLB sample obtained in the buffer solution without CHO-PL at (a) 25 °C and (b) 37 °C. Scale bar = 20 μm.

Thickness of PC+PE+Chol-SLB.

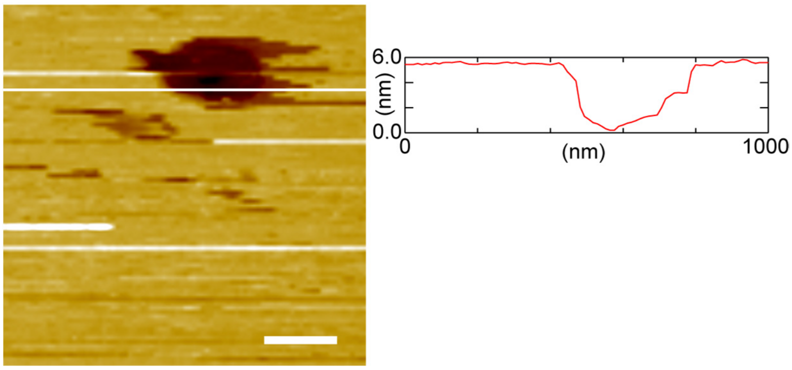

Figure S2. AFM topography of PC+PE+Chol-SLB with a defect in a depression domain. AFM topography (1.0 × 1.0 μm^2) obtained after the cantilever was compressed to SLB, and the cross section profile at the white line. Scale bar = 200 nm.

Estimation of approaching frequency of PL to SLB.

The diffusion constant (D) of PL ~400 nm in diameter (d) in a bulk aqueous solution at 37 °C is estimated to be 1.6 μm^2/s based on the Stokes-Einstein equation, $D=k_BT/3\pi\eta d$, where k_B, T and η represent the Boltzman constant, temperature and dynamic viscosity, respectively. One dimensional average diffusion length (l) along Z axis is $l=\sqrt{2Dt}$ =1.8 μm at t=1 s. The CHO-PL was a crude membrane fraction containing 0.038 mg/mL of protein. The ratio of proteins to lipids in most membranes is ~1:1 by weight, therefore we may evaluate the density of CHO-PL (d=400 nm) to be 3.8×10^{-2} μm^{-3}, assuming that molecular mass and occupying area of lipid are ~800 g/mol and 0.6 nm^2 per lipid molecule (Nagle, J. F. & Tristram-Nagle, S. *Biochim. Biophys. Acta* **1469**, 159–195 (2000)), respectively. Half of PL in the region of l (=1.8 μm)×1 μm×1 μm nearby SLB diffuse downward, hence the approaching frequency of PL to SLB is estimated to be 2.3×10^{-2} PL μm^{-2} s^{-1}, which corresponds that 80 PLs approach to 1 μm^2 of SLB during 60 min.

Growth of PL domains depending on kinetic constant of PL fusion ($\boldsymbol{k_f}$).

Movie S2. Time course of the kinetic simulation with $k_f = 0.0002$, from which Figs. 7b-1 to 7b-5 were obtained. The step number of the calculation is inserted.

Movie S3. Time course of the kinetic simulation with $k_f = 0.02$, from which Figs. 7c-1 and 7c-2 were obtained. The step number of the calculation is inserted.
